# Plant–Soil–Microbe Interactions Along a Salinity Gradient in the Songnen Plain Grasslands

**DOI:** 10.3390/microorganisms14040860

**Published:** 2026-04-11

**Authors:** Haotian Li, Wenbo Zhu, Tianen Hu, Yilin Chen, Zhihao Han, Huichuan Xiao, Ligang Qin, Linlin Mei

**Affiliations:** College of Animal Science and Technology, Northeast Agricultural University, Harbin 150030, China

**Keywords:** salinization, saline–alkali grassland, structural equation modeling, microbial diversity, plant diversity, soil physicochemical properties

## Abstract

The salinization of natural grasslands is a growing global concern. The Songnen Plain in northeastern China represents a typical soda–saline grassland region, yet an integrated understanding of how salinization reshapes plant, soil, and microbial components in this ecosystem remains limited. In this study, we investigated plant community characteristics, soil physicochemical properties, and soil microbial communities across a salinity gradient (from non-saline to extremely severe saline) using field surveys, laboratory analyses, and structural equation modeling (SEM). Our results showed that vegetation species diversity, the Shannon–Wiener index, and Simpson’s index all decreased from mild to severe salinization. Soil nutrient indicators, including total nitrogen (TN), total phosphorus (TP), and total potassium (TK), significantly decreased with increasing salinity. SEM revealed that plant community diversity had a significant positive effect on soil microorganisms, whereas soil properties, particularly available potassium (AK) and electrical conductivity (EC), exerted significant negative effects on microbial diversity. Together, these results provide an integrated view of how salinization restructures plant–soil–microbe interactions across the Songnen Plain grasslands. These findings improve understanding of saline–alkali grassland degradation from a plant–soil–microbe perspective and provide a theoretical basis for ecosystem restoration in this region.

## 1. Introduction

Natural ecosystems are highly complex and are shaped by the combined effects of abiotic and biotic factors, including climate, environmental heterogeneity, disturbance, and biological interactions, all of which jointly influence community composition and ecosystem diversity [1,2]. Among the major environmental constraints affecting terrestrial ecosystems, soil salinization is one of the most widespread forms of land degradation [3]. It is generally caused by the accumulation of soluble salts in the soil profile or root zone, which may result from natural processes such as strong evaporation, shallow groundwater movement, and salt-rich parent materials, as well as human activities such as improper irrigation and poor drainage [3,4].

In grassland ecosystems, increasing salinity can impose osmotic stress and ion toxicity on plants, thereby reducing plant growth, altering species composition, and constraining aboveground productivity [5]. At the same time, salinization can modify soil physicochemical conditions, including electrical conductivity, pH, and nutrient availability, and may further suppress microbial activity, nutrient cycling, and organic matter turnover [4,5]. Because plant communities regulate the quantity and quality of carbon inputs to soil, while soil microorganisms respond sensitively to salinity-induced shifts in physicochemical conditions, salinization is expected to reshape plant–soil–microbe interactions rather than affect plants, soils, and microorganisms independently [2,5,6].

A critical concept for understanding these linkages is plant–soil feedback (PSF), first introduced by Bever [7]. PSF refers to the process by which plants modify soil physicochemical properties and microbial communities, which in turn influence the performance of the same or subsequent plant species, thereby affecting ecosystem structure and function. Previous studies have shown that PSF can influence plant performance, species coexistence, community composition, and successional dynamics by linking plant-induced changes in soil biotic and abiotic conditions to subsequent plant responses [8,9]. The interactions between plants, soils, and microorganisms are therefore central to these feedback mechanisms [2]. Soil microorganisms are vital components of soil ecosystems because they participate in most biogeochemical transformations and strongly influence nutrient availability, plant performance, and ecosystem resilience [9]. Plants can also influence soil microbial communities indirectly through root exudates and litter inputs, thereby driving shifts in microbial structure and function [10]. This framework is particularly relevant in saline–alkali grasslands, where salinity stress can simultaneously affect plant community composition, soil physicochemical properties, and microbial assemblages, thereby potentially modifying the strength and direction of plant–soil feedback [4,5].

Despite the extensive attention paid to plant–soil feedback and plant–microbe interactions in studies of ecosystem functioning and succession [8,9], it remains unclear how plant diversity, salinization-related soil physicochemical properties, and microbial diversity co-vary across saline–alkali degradation gradients, and to what extent their relationships are driven by direct versus indirect interactions under saline–alkali stress [4,5]. This issue is particularly relevant in the Songnen Plain of Northeast China, which is recognized as one of the most representative soda saline–alkali regions in the world and where saline–alkali degradation strongly affects vegetation composition, soil properties, and microbial diversity [11,12]. However, the mechanisms governing the co-variation in plant, soil, and microbial factors across salinity gradients in this region are still not fully resolved.

In this study, we investigated plant–soil–microbe interactions across a salinity-induced degradation gradient in the typical saline–alkali meadow grasslands of the Songnen Plain, Northeast China. We compared plant biomass, soil physicochemical properties, and soil microbial diversity among five grassland stages representing increasing degrees of saline–alkali degradation, and used structural equation modeling to evaluate the direct and indirect relationships among plant, soil, and microbial variables. We hypothesized that increasing saline–alkali degradation would be associated with declines in plant diversity and biomass, deterioration of soil physicochemical conditions, and shifts in soil microbial diversity and community structure. We further hypothesized that plant diversity and salinization-related soil properties would jointly regulate microbial variation along this gradient. Our objective was to clarify the feedback mechanisms underlying plant–soil–microbe interactions along this gradient and to provide a theoretical basis for the restoration of degraded saline grasslands.

## 2. Materials and Methods

### 2.1. Site Description and Experimental Design

The research was conducted at the Suihua Station of the Chinese National Technology System of Forage Industry (125°28′24″ E, 46°32′17″ N), located in the southeastern Songnen Plain, China. This region typically experiences a continental monsoon climate. According to meteorological records from the local meteorological station, the average annual precipitation is 469.7 mm, the average annual temperature is 2.9 °C, the annual accumulated temperature (≥10 °C) is 2760 °C, and the annual sunshine duration is 2713 h. The frost-free period is approximately 130 days, and the annual freezing period lasts for 183 days. The soil type is primarily characterized as light chernozem and salinized meadow soil according to the Chinese soil classification system. Vegetation in the area is dominated by mesophytic or xerophytic gramineous plants. The community is dominated by *Leymus chinensis* (Trin.) Tzvel., accompanied by species such as *Lathyrus quinquenervius* (Miq.) Litv., *Chloris virgata* Sw., *Puccinellia distans* (L.) Parl., *Hemarthria altissima* (Poir.) Stapf et C. E. Hubb., *Carex duriuscula* subsp. *stenophylloides* (V. I. Kreczetowicz) S. Yun Liang & Y. C. Tang, *Phragmites communis*, and *Suaeda heteroptera* Kitag.

Five grassland stages representing increasing degrees of saline–alkali degradation were identified based on vegetation community composition together with salinity-related soil characteristics: (1) mild salinity grassland (MIS), dominated by Leymus chinensis; (2) moderately saline grassland (MDS), dominated by miscellaneous grasses; (3) heavily saline grassland (HES), dominated by *Puccinellia tenuiflora*; (4) extremely severe saline grassland (ETS), dominated by *Suaeda heteroptera*; and (5) alkali spots (AS), which were devoid of vegetation. In each site, three 10 m × 10 m replicate plots were established with a 1 m buffer interval. This plot size was selected to represent the community-level heterogeneity of grassland vegetation at each salinity stage. Within each plot, four 1 m × 1 m quadrats were randomly arranged to record species composition, coverage, plant height, density, and above-ground biomass. The quadrat size and number were chosen to obtain representative measurements of herbaceous community characteristics while accounting for within-plot spatial variation.

Soil samples were collected from three depths (0–10 cm, 10–20 cm, and 20–30 cm) in each plot using a five-point sampling method. These depth intervals were selected to characterize the vertical variation in topsoil properties within the main rooting zone of grassland vegetation. Samples from the same layer were thoroughly mixed after removing stones and plant residues. The composite samples were divided into two portions: one was air-dried in the laboratory for physicochemical analysis, and the other was stored in sterile 50 mL centrifuge tubes, transported on dry ice, and stored at −80 °C for microbial analysis. In total, 45 composite soil samples were obtained for subsequent soil physicochemical analyses and microbial community analyses.

### 2.2. Plant Community Measurements

The number of species in each quadrat was recorded. Above-ground biomass was determined by clipping the vegetation at ground level, drying it at 65 °C to a constant weight, and weighing it. The natural height of each plant species was measured (taking the average of three measurements per species). Plant coverage was estimated visually, and the density was recorded as the number of individuals per species. The Shannon–Wiener index (H′) and Simpson’s index (D) were calculated as follows:

(1) $$H^{\prime }\;=\;-\sum P_{i}\ln P_{i}$$

(2) $$D=1-\sum P_{i}^{2}$$

where *P_i_* is the proportion of individuals of species *i* relative to the total number of individuals in the community.

### 2.3. Soil Properties Determination

Soil physicochemical properties were determined using standard procedures described in previous studies [13]. Soil pH was measured in a 1:2.5 soil–water suspension using a pH meter, and electrical conductivity (EC) was determined using a conductivity meter in the same extract. Soil water content (SWC) was measured gravimetrically by drying samples at 105 °C overnight. Alkaline hydrolyzable nitrogen (AN) was measured using the alkaline diffusion method. Total potassium (TK) and available potassium (AK) were determined by flame photometry after sodium hydroxide fusion and ammonium acetate extraction, respectively. Total phosphorus (TP) was determined by the molybdenum–antimony colorimetric method after sodium hydroxide fusion. Total nitrogen (TN) was determined using the Kjeldahl method [14]. Soil organic matter (SOM) was determined by potassium dichromate oxidation following Walkley and Black [15]. Available phosphorus (AP) was measured after sodium bicarbonate extraction according to Olsen [16]. In this study, the term “soil properties” refers collectively to the measured soil physicochemical and nutrient variables, including pH, EC, SWC, SOM, TN, TP, TK, AN, AK, and AP.

### 2.4. Microbial Abundance and Community Composition

Total genomic DNA was extracted from 0.5 g of fresh soil using the DNeasy PowerSoil DNA Isolation Kit (MoBio Laboratories, Carlsbad, CA, USA) according to the manufacturer’s instructions. DNA integrity was assessed by 1% agarose gel electrophoresis, and DNA concentration was quantified using a Qubit 2.0 DNA Assay Kit (Life Technologies, Carlsbad, CA, USA). The V4 region of the bacterial 16S rRNA gene was amplified using primers 515F (5′-GTGCCAGCMGCCGCGGTAA-3′) and 806R (5′-GGACTACHVGGGTWTCTAAT-3′), while the fungal ITS1 region was amplified using primers ITS1F (5′-CTTGGTCATTTAGAGGAAGTAA-3′) and ITS2 (5′-GCTGCGTTCTTCATCGATGC-3′). PCR reactions were performed in 30 μL volumes containing 15 μL of Phusion^®^ High-Fidelity PCR Master Mix (New England Biolabs, Ipswich, MA, USA), 0.2 μM of each primer, and approximately 10 ng of template DNA. Thermal cycling conditions consisted of an initial denaturation at 98 °C for 1 min, followed by 30 cycles of denaturation at 98 °C for 10 s, annealing at 50 °C for 30 s, and extension at 72 °C for 30 s, with a final extension at 72 °C for 5 min. Negative controls were included during both DNA extraction and PCR amplification to monitor potential contamination. PCR products were verified on 2% agarose gels, purified using the Qiagen Gel Extraction Kit (Qiagen, Germany), and sequenced on an Illumina MiSeq platform (Illumina, San Diego, CA, USA) using a 2 × 250 bp paired-end protocol. Raw sequences were processed using the QIIME 2 pipeline (v2022.2). Reads were demultiplexed and quality-filtered with the DADA2 plugin to generate amplicon sequence variants (ASVs), and chimeric sequences were removed. Taxonomic assignment was conducted against the SILVA database (release 138) for bacteria and the UNITE database (release 8.2) for fungi. To standardize sequencing depth across samples, the feature table was rarefied to the minimum sequencing depth prior to diversity analyses.

### 2.5. Statistical Analyses

All data analyses were performed using R software (version 4.3.1). One-way analysis of variance (ANOVA) followed by Tukey’s honestly significant difference (HSD) test was used to compare plant biomass, diversity indices, and soil physicochemical properties across the different salinity gradients. Alpha diversity indices (Shannon–Wiener and Simpson) were calculated using the ‘vegan’ package. Principal Component Analysis (PCA) was applied to the genus-level relative abundance matrix to summarize and visualize major patterns of variation in microbial community composition among the salinity gradients. Genera with significantly different abundances between groups were identified using the ‘DESeq2’ package. Canonical Correspondence Analysis (CCA) was applied to evaluate the relationships between microbial community structure and environmental variables. Additionally, Structural Equation Modeling (SEM) was constructed using the ‘lavaan’ package to quantify the direct and indirect pathways driving plant–soil–microbe interactions. Differences were considered statistically significant at *p* < 0.05.

## 3. Results

### 3.1. Analysis of Plant Community Diversity and Soil Properties in Different Salinity Gradients

**Figure 1:**
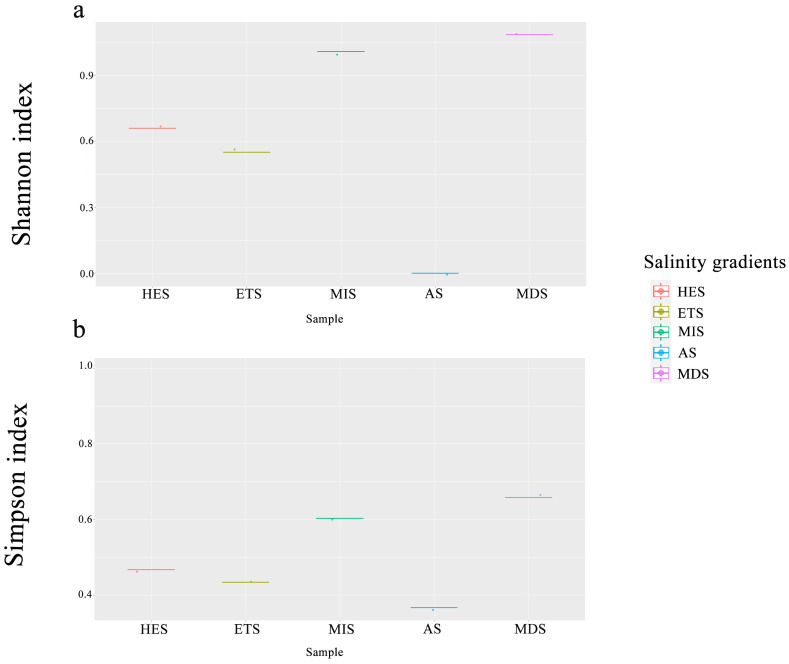
Shannon–Wiener index (**a**) and Simpson index (**b**) of plant alpha diversity in grasslands with different salinity gradients. HES: heavily saline grassland, ETS: extremely severe saline grassland, MIS: mild salinity grassland, AS: alkali spot, MDS: moderately saline grassland.

Soil pH increased significantly across the salinity gradient, with lower values in MIS and MDS and higher values in HES, ETS, and AS (Figure S1). Plant alpha diversity showed the opposite pattern, generally decreasing from the lower-salinity stages to the more severely salinized stages. Specifically, the Shannon–Wiener index was highest in the MDS (1.08) and lowest in the AS (0.55) (Figure 1a). Similarly, the Simpson index was highest in the MDS and lowest in the AS (Figure 1b). Alpha diversity in ETS and HES did not differ significantly from each other, but both were significantly lower than that in MIS and MDS.

Soil properties varied across salinity gradients and soil depths. Soil pH increased with increasing salinity and soil depth, whereas SWC and SOM generally decreased with increasing salinity. Nutrient variables showed contrasting patterns along the salinity gradient: TN, TK, and TP generally declined with increasing salinity, whereas AN, AK, and AP generally increased. Within the same soil depth, all measured soil physicochemical properties showed significant differences among salinity stages, as indicated by the lowercase letters in Figure S1. Across the three soil layers, AN, AK, and AP tended to be higher in the 10–20 cm layer.

### 3.2. Composition and Diversity of Soil Bacterial Communities

**Figure 2:**
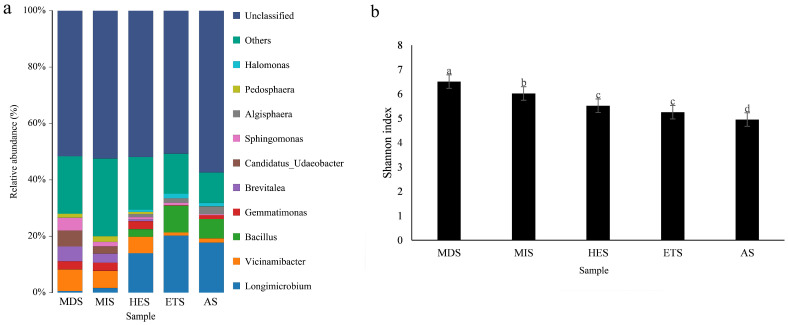
Histogram of soil bacterial species distribution (**a**) and Shannon index of bacterial diversity (**b**) on grasslands with different salinization gradients. MIS: mild salinity grassland, MDS: moderately saline grassland, HES: heavily saline grassland, ETS: extremely severe saline land, AS: alkali spot. Different lowercase letters indicate significant differences among groups (*p* < 0.05).

Bacterial community composition varied across the salinity gradient. Among the classified genera, *Longimicrobium* increased from 0.64% in MDS and 1.56% in MIS to 12.93% in HES, 20.33% in ETS, and 17.84% in AS. *Bacillus* showed a similar pattern, with relative abundance increasing from 0.07% in MDS and 0.05% in MIS to 2.70% in HES, 9.02% in ETS, and 6.71% in AS. In contrast, *Brevitalea* declined from 5.28% in MDS and 3.31% in MIS to 0.77% in HES and was nearly absent in ETS and AS. *Vicinamibacter* was relatively abundant in MDS (7.62%), MIS (6.26%), and HES (6.06%), but much lower in ETS (1.21%) and AS (1.48%). *Gemmatimonas* showed relatively higher abundance in MDS (2.70%), MIS (2.74%), and HES (3.00%), but lower abundance in ETS (0.21%) and AS (1.39%) (Figure 2a). Bacterial alpha diversity also differed among the salinity stages. The Shannon–Wiener index was highest in the MDS and lowest in the ETS, and significant differences in bacterial diversity were observed among the salinity gradients (Figure 2b).

**Figure 3:**
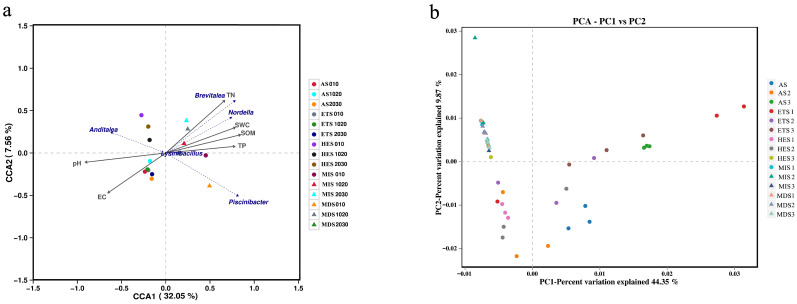
Soil bacteria on grasslands with different salinization gradients, canonical correlation analysis (CCA) (**a**) and principal component analysis (PCA) (**b**) of soil physicochemical properties. MIS: mild salinity grassland, MDS: moderately saline grassland, HES: heavily saline grassland, ETS: extremely severe saline land, AS: alkali spot.

The CCA results showed that the first axis explained 32.05% of the variation, whereas the second axis explained 7.56% (Figure 3a). Most MIS and MDS samples were distributed on the positive side of CCA1, while AS, ETS, and HES samples were mainly located on the negative side. Soil TN, SWC, SOM, and TP were oriented toward the positive side of CCA1, whereas pH and EC were oriented toward the negative side. Accordingly, MIS and MDS were more closely associated with higher TN, SWC, SOM, and TP, whereas AS, ETS, and HES were more closely associated with higher pH and EC. Among the bacterial genera, *Brevitalea* and *Nordella* were aligned with TN, SWC, and SOM, whereas *Anditalea* was distributed toward the negative side of CCA1. *Piscinibacter* was positioned on the positive side of CCA1 and the negative side of CCA2, while *Lysinibacillus* was located near the origin (Figure 3a).

PCA showed that the first principal component (PC1) and the second principal component (PC2) explained 44.35% and 9.87% of the variation, respectively (Figure 3b). MDS and MIS samples were mainly clustered on the negative side of PC1 and showed considerable overlap. In contrast, ETS samples were clearly separated on the positive side of PC1, indicating a distinct bacterial community structure under more severe salinization. AS samples were also mainly distributed on the positive side of PC1, whereas HES samples were primarily located on the negative side of PC1 and the negative side of PC2. Overall, the PCA revealed clear shifts in bacterial community structure along the salinity gradient, with strong overlap between MDS and MIS and marked separation of ETS from the other stages (Figure 3b).

### 3.3. Composition and Diversity of Soil Fungal Communities

**Figure 4:**
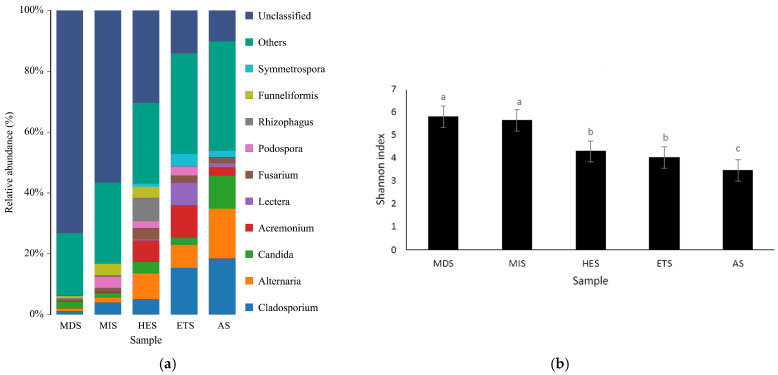
Histogram of soil fungal species distribution (**a**) and Shannon index of fungal diversity (**b**) on grasslands with different salinization gradients. MIS: mild salinity grassland, MDS: moderately saline grassland, HES: heavily saline grassland, ETS: extremely severe saline land, AS: alkali spot. Different lowercase letters indicate significant differences among groups (*p* < 0.05).

Fungal community composition varied markedly across the salinity gradient. The relative abundance of unclassified fungi decreased from the lower-salinity stages to the more severely salinized stages, whereas the proportion of the “Others” category increased in ETS and AS. Among the classified genera, *Cladosporium* increased from 1.24% in MDS and 4.12% in MIS to 5.16% in HES, 15.76% in ETS, and 18.69% in AS. *Alternaria* showed a similar pattern, increasing from 0.92% in MDS and 1.71% in MIS to 8.55% in HES, 7.34% in ETS, and 16.88% in AS. *Candida* was also more abundant in the more severely salinized stages, with 2.04% in MDS, 1.57% in MIS, 3.64% in HES, 2.70% in ETS, and 10.07% in AS. In contrast, *Acremonium* was most abundant in HES (6.80%) and ETS (10.46%), whereas *Funneliformis*, *Rhizophagus*, and *Podospora* showed relatively higher abundances in MIS and HES than in ETS and AS. Specifically, *Funneliformis* accounted for 0.69% in MDS, 3.30% in MIS, and 3.55% in HES, but was nearly absent in ETS and AS; *Rhizophagus* accounted for 0.25% in MDS, 0.42% in MIS, and 7.57% in HES, but was absent or nearly absent in ETS and AS; and *Podospora* accounted for 0.15% in MDS, 3.37% in MIS, 2.28% in HES, and 2.80% in ETS, but only 0.02% in AS (Figure 4a). Fungal alpha diversity also differed among the salinity stages. The Shannon index decreased with increasing salinity, with the highest value observed in MDS (5.089) and the lowest in AS (3.457) (Figure 4b). MDS and MIS showed higher fungal diversity than HES and ETS, whereas AS had the lowest diversity.

**Figure 5:**
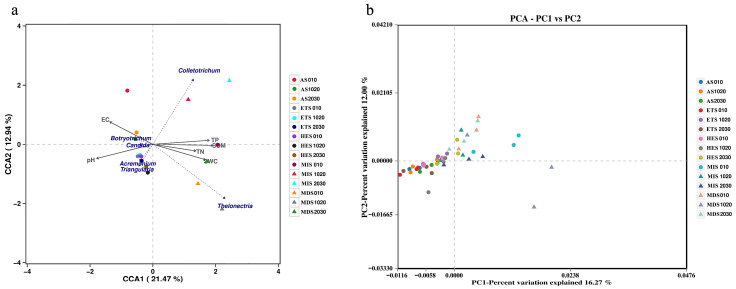
Soil fungi on grasslands with different salinization gradients, canonical correlation analysis (CCA) (**a**) and principal component analysis (PCA) (**b**) of soil physicochemical properties. MIS: mild salinity grassland, MDS: moderately saline grassland, HES: heavily saline grassland, ETS: extremely severe saline land, AS: alkali spot.

The CCA results showed that the first axis explained 21.47% of the variation, whereas the second axis explained 12.94% (Figure 5a). Most MIS and MDS samples were distributed toward the positive side of CCA1, whereas AS, ETS, and HES samples were more closely associated with the negative side. Soil TP, SOM, TN, and SWC were oriented toward the positive side of CCA1, whereas pH and EC were oriented toward the negative side. Among the fungal genera, *Colletotrichum* and *Thelonectria* were positioned on the positive side of CCA1, while *Acremonium* and *Triangularia* were distributed toward the negative side of CCA1. *Botryotrichum* and *Candida* were located closer to the negative side of the ordination space (Figure 5a).

PCA showed that the first principal component (PC1) and the second principal component (PC2) explained 16.27% and 12.00% of the variation, respectively (Figure 5b). Most MIS and MDS samples were distributed on the positive side of PC1, whereas AS, ETS, and HES samples were mainly located on the negative side of PC1 or close to the origin. Some overlap among the salinity stages was still evident, particularly among the more severely salinized stages. Overall, the PCA indicated variation in fungal community structure across the salinity gradient, although the separation among stages was less distinct than that observed for the bacterial communities (Figure 5b).

### 3.4. Structural Equation Modeling of Plant–Soil–Microbe Interactions

**Figure 6:**
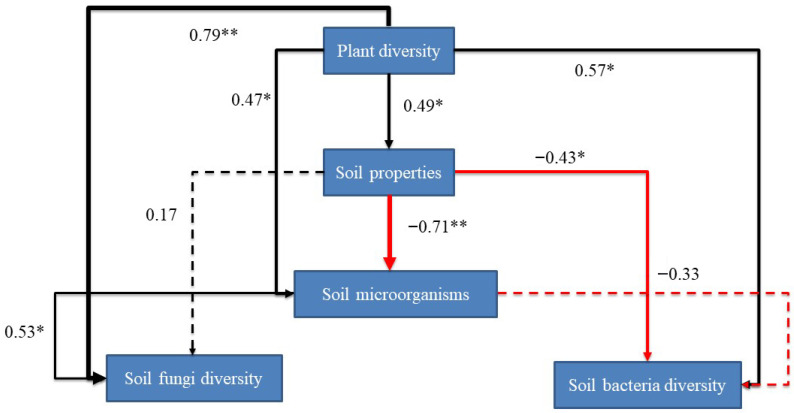
Structural equation modeling (SEM) of plant–soil–microbe interrelationships. Different lowercase letters indicate significant differences among groups. * *p* < 0.05; ** *p* < 0.01.

The final SEM showed an adequate fit to the data describing the interaction pathways among plant, soil, and microbial components (χ^2^ = 13.473, df = 13, *p* = 0.412; standardized path coefficients are shown in Figure 6). Plant diversity had a significant positive effect on soil properties (0.49, *p* < 0.05), soil microorganisms (0.47, *p* < 0.05), soil fungi diversity (0.79, *p* < 0.01), and soil bacterial diversity (0.57, *p* < 0.05). In contrast, soil properties had a significant negative effect on soil microorganisms (−0.71, *p* < 0.01) and on soil bacterial diversity (−0.43, *p* < 0.05).

Soil microorganisms also had a significant positive effect on soil fungi diversity (0.53, *p* < 0.05). In addition, soil properties showed a weak positive path to soil fungi diversity (0.17), whereas soil microorganisms showed a negative path to soil bacterial diversity (−0.33); however, these two paths were not significant. Overall, the SEM revealed both direct and indirect linkages among plant diversity, soil properties, and microbial components across the salinity gradient (Figure 6).

## 4. Discussion

Salinity-induced degradation in the Songnen Plain simultaneously altered plant community diversity, soil physicochemical properties, and belowground microbial communities. In this study, the lower-salinity stages were characterized by higher plant diversity, more favorable soil conditions, and greater bacterial and fungal diversity, whereas the more severely salinized stages were associated with higher pH and EC, reduced plant diversity, and clear shifts in microbial community composition. The SEM results further indicated that plant diversity and soil properties jointly shaped microbial variation, highlighting that plant–soil–microbe interactions along the salinity gradient were regulated by both soil environmental filtering and plant community change. These findings provide an integrated framework for understanding how saline–alkali degradation restructures aboveground and belowground biotic linkages in the Songnen Plain.

Soil properties are critical indicators of community organization, structure, and succession, playing a pivotal role in grassland ecosystems [17,18]. Plant biomass is a key factor for assessing grassland ecosystems and is closely linked to both community structure and soil properties [19,20]. In the present study, plant diversity varied clearly across the salinity gradient, with the highest alpha diversity observed in the miscellaneous grass community (MDS) and the lowest in the alkali spot stage (AS). This pattern was accompanied by an increase in soil pH and a decline in SWC along the salinity gradient, indicating that increasing saline–alkali stress was associated with reduced plant diversity in the Songnen Plain. Rather than implying a simple one-way causal relationship, these results suggest that soil moisture limitation and increasing alkalinity were closely linked to the observed reduction in plant community diversity [21,22].

This study demonstrates that SWC and pH are crucial drivers of plant community diversity in the Songnen Plain. Our results are also consistent with previous studies showing that pH and water availability are among the principal environmental factors regulating plant establishment, competitive balance, and community turnover in grassland ecosystems [23,24,25]. Under saline–alkali conditions, elevated pH and reduced water availability may constrain seedling establishment, nutrient uptake, and the persistence of less stress-tolerant species, thereby favoring simplified plant assemblages under severe salinization [21,26,27].

Furthermore, physicochemical indicators such as TK and AN significantly affect the diversity and biomass of different plant communities [28]. Our results support the view that the degree of salinization in the Songnen Plain is closely related to plant community composition [11,29,30]. In this study, soil nutrients exerted a substantial influence on plant biomass and diversity. Since vegetation growth relies heavily on soil nutrient supply and fertility, a reciprocal relationship exists between them. The results from Figure S1 further indicate that nutrient variables did not change uniformly along the salinity gradient: TN, TK, and TP generally declined with increasing salinity, whereas AN, AK, and AP tended to increase. This pattern suggests that saline–alkali degradation in the Songnen Plain is not only a gradient of increasing salt stress, but also a gradient of changing soil resource availability. Such shifts in nutrient availability may contribute to the replacement of diverse grassland communities by salt-tolerant and stress-adapted vegetation types [29,30]. Consequently, changes in soil fertility and nutrient status are intrinsic drivers of the degradation processes observed in the Songnen Plain [29,31,32].

These aboveground and soil changes were further mirrored belowground by pronounced shifts in microbial diversity and community composition. As the most active component of the ecosystem, soil microorganisms play a vital role in energy flow, nutrient cycling, and plant growth. Consequently, microbial diversity serves as a key indicator for evaluating ecosystem stability [4,33,34]. In the present study, soil microbial diversity varied across the salinity gradient, with the highest values observed in the miscellaneous grass community (MDS) and lower diversity in the more severely salinized stages. In addition, both bacterial and fungal community structures varied along the gradient, indicating that salinity-induced degradation was accompanied by changes in belowground microbial assemblages [11,12].

Soil acts as the most direct factor influencing microbial diversity, which typically increases with soil nutrient availability [35,36]. Numerous studies have identified soil pH and SWC as primary drivers of microbial diversity, and the results of this study confirm this general pattern [37,38,39]. Comparable evidence from subtropical forests has also shown that vegetation type, seasonal variation, and soil properties can jointly shape microbial community structure across environmental gradients [39]. Our bacterial CCA results further showed that the lower-salinity stages (MIS and MDS) were more closely associated with higher TN, SWC, SOM, and TP, whereas the more severely salinized stages (AS, ETS, and HES) were more closely associated with higher pH and EC. At the genus level, *Brevitalea* and *Nordella* were enriched in low-salinity stages, whereas *Anditalea* was associated with high-salinity conditions. These results suggest that salinity-related shifts in soil properties acted as environmental filters that restructured bacterial communities across the gradient [12,36]. Such a pattern is consistent with previous studies showing that increasing salinity and alkalinity can reduce microbial diversity and alter community composition through changes in EC, pH, and nutrient status [40,41].

However, soil microbial diversity in the Songnen Plain grasslands is not solely governed by edaphic properties; it is also significantly shaped by the plant community [11,36,42]. Recent synthesis evidence further supports this interpretation, showing that soil microbiota can exert broad effects on both plant performance and soil processes across ecosystems [42]. Our findings indicate that microbial diversity peaked in the miscellaneous grass community (MDS), corresponding to a moderately degraded grassland stage. This observation aligns with previous studies suggesting that intermediate disturbance or degradation levels may support higher diversity [43,44]. The SEM results further support this interpretation: plant diversity exerted significant positive effects on soil microorganisms (0.47, *p* < 0.05), soil fungal diversity (0.79, *p* < 0.01), and soil bacterial diversity (0.57, *p* < 0.05). In addition, soil microorganisms had a significant positive effect on soil fungal diversity (0.53, *p* < 0.05). These results indicate that plant community change is not simply a passive response to salinity, but also an important biological driver of belowground microbial organization.

Changes in plant community composition may influence microbial communities through shifts in litter input, rhizodeposition, and the quantity and quality of soil organic substrates. Previous studies have shown that plant-derived carbon inputs, particularly root-derived inputs, are important regulators of soil microbiomes and associated ecosystem functions [10,11]. As vegetation succession proceeds, plant species composition shifts and plant diversity increases [8], leading to concurrent changes in litter, root exudates, and soil organic matter—the primary carbon substrates for soil microbes. These changes subsequently drive significant shifts in soil microbial community structure and enhance microbial species diversity [11,43,44,45].

Fungal communities showed particularly clear responses to the salinity gradient. In our results, fungal Shannon diversity declined from the lower-salinity stages to the more severely salinized stages, and fungal community composition also changed substantially. Genera such as *Alternaria* and *Candida* were more abundant in the more severely salinized stages, whereas *Funneliformis*, *Rhizophagus*, and *Podospora* showed relatively higher abundances in the lower-salinity or intermediate stages. The fungal ordination results further indicated that community variation was associated with the same salinity-related environmental gradient, with pH and EC aligned with the more severely salinized stages and TN, SWC, SOM, and TP aligned with the lower-salinity stages. These results suggest that fungal assemblages were strongly structured by saline–alkali environmental filtering [4,46]. Some of the enriched genera in the more severely salinized stages include taxa reported in the literature as plant-associated or potentially pathogenic fungi; however, because the present study was based on community composition rather than direct functional assignment, this ecological implication should be interpreted cautiously [40]. Future metagenomic or culture-based studies are warranted to clarify their functional roles under saline conditions.

Furthermore, microhabitat heterogeneity and variations in soil organic matter have been shown to significantly impact the distribution of soil microbial communities [11,47,48]. Taken together, our results indicate that microbial variation along the salinity gradient in the Songnen Plain was jointly regulated by soil environmental filtering and plant community change, rather than by a single factor alone. Beyond their role in community turnover and nutrient cycling, soil microorganisms also represent an important functional component of ecosystem processes, with broader implications for soil health and biochemical transformation under environmental stress [49].

## 5. Conclusions

In this study, we investigated variation in plant community diversity, soil properties, and soil microbial diversity, as well as their interrelationships, across a salinity gradient in the Songnen Plain grasslands. Our results showed that salinity-induced degradation progressively altered plant–soil–microbe linkages, with reduced plant diversity, less favorable soil conditions, and clear shifts in microbial community structure under the more severely salinized stages. Notably, both plant biomass and soil microbial diversity were highest in the moderately saline vegetation community (MDS). Structural equation modeling further indicated that plant diversity exerted positive effects on microbial diversity, whereas salinization-related soil physicochemical conditions acted as important constraints on microbial variation. These findings suggest that ecological restoration of salinized grasslands in the Songnen Plain should prioritize the regulation of plant community composition and the amelioration of soil physicochemical properties in order to reduce nutrient loss, enhance community stability, and promote the coordinated recovery of plant and microbial diversity. Although this study provides an integrated view of plant–soil–microbe interactions along the salinity gradient, it was mainly based on community composition, diversity patterns, and structural relationship analyses, and therefore did not directly resolve microbial functional traits or causal mechanisms. Future studies should combine metagenomics, culture-based approaches, and long-term field monitoring to further clarify the functional roles of soil microorganisms and the mechanistic basis of plant–soil–microbe feedback under saline–alkali stress.

## Data Availability

The original contributions presented in this study are included in the article/Supplementary Material. Further inquiries can be directed to the corresponding authors.

## Supplementary Materials

The following supporting information can be downloaded at: https://www.mdpi.com/article/10.3390/microorganisms14040860/s1, Figure S1: Variations in soil physicochemical properties across different salinity gradients.
