# Supplementary material for: Plant–Soil–Microbe Interactions Along a Salinity Gradient in the Songnen Plain Grasslands
Source: Microorganisms. 2026 Apr 11;14(4):860. doi: 10.3390/microorganisms14040860 (PMC13118986; doi:10.3390/microorganisms14040860)
Supplement: Supplementary file 1 [file microorganisms-14-00860-s001.zip › microorganisms-4179378-supplementary.pdf]

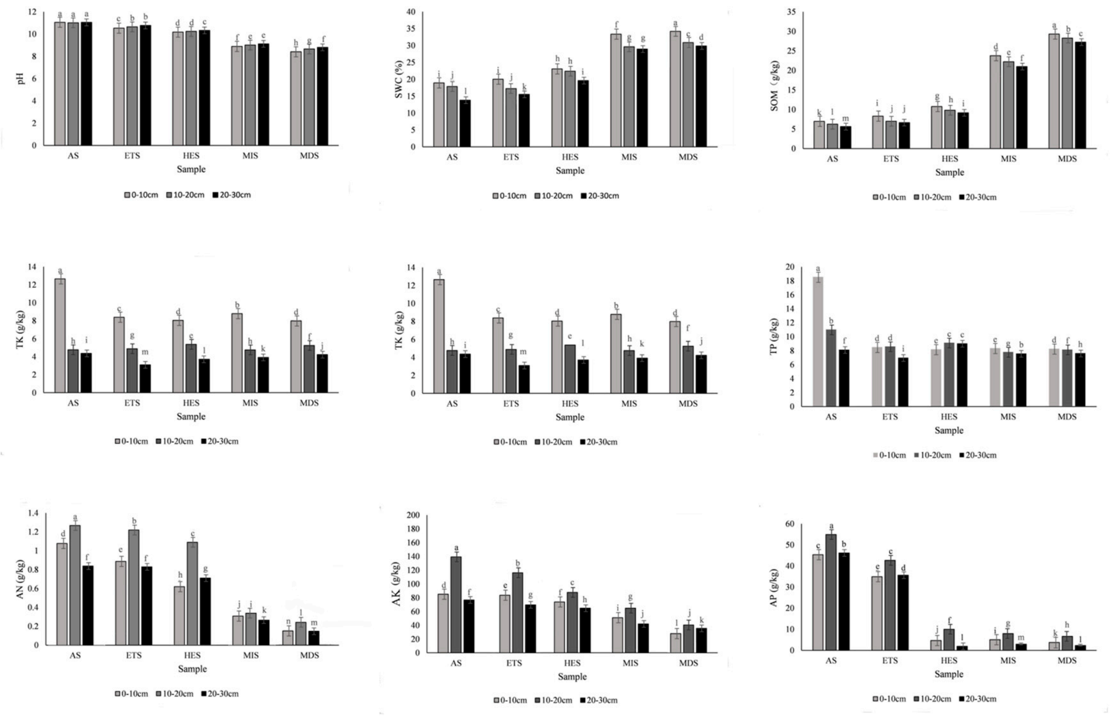

Figure S1. Variations in soil physicochemical properties across salinity gradients and soil depths. Bar charts show mean values of soil properties including pH, soil water content (SWC), soil organic matter (SOM), total potassium (TK), total nitrogen (TN), total phosphorus (TP), alkaline nitrogen (AN), available potassium (AK), and available phosphorus (AP). Data are presented as mean ± standard error (SE). Different lowercase letters above the bars indicate significant differences among salinity gradients within the same soil depth ($P < 0.05$). Abbreviations: MIS, mild salinity grassland; MDS, moderately saline grassland; HES, heavily saline grassland; ETS, extremely severe saline grassland; AS, alkali spots.
